# Exploring metabolism in scleroderma reveals opportunities for pharmacological intervention for therapy in fibrosis

**DOI:** 10.3389/fimmu.2022.1004949

**Published:** 2022-10-11

**Authors:** Isabella Gomes Cantanhede, Huan Liu, Huan Liu, Vestaen Balbuena Rodriguez, Xu Shiwen, Voo H. Ong, Christopher P. Denton, Markella Ponticos, Guo Xiong, José Luiz Lima-Filho, David Abraham, Jeries Abu-Hanna, Jan-Willem Taanman

**Affiliations:** ^1^ Centre for Rheumatology and Connective Tissue Diseases, Division of Medicine, University College London, London, United Kingdom; ^2^ Laboratory of Immunopathology Keizo Asami, Federal University of Pernambuco, Recife, Brazil; ^3^ Health Science Center, Xi’an Jiaotong University, Xi’an, China; ^4^ Department of Clinical and Movement Neurosciences, Queen Square Institute of Neurology, University College London, London, United Kingdom

**Keywords:** fibrosis, glycolysis, kinase inhibitors, mitochondrial morphology, mitochondrial respiration, myofibroblasts, oxidative phosphorylation, systemic sclerosis

## Abstract

**Background:**

Recent evidence has indicated that alterations in energy metabolism play a critical role in the pathogenesis of fibrotic diseases. Studies have suggested that ‘metabolic reprogramming’ involving the glycolysis and oxidative phosphorylation (OXPHOS) in cells lead to an enhanced generation of energy and biosynthesis. The aim of this study was to assess the molecular basis of changes in fibrotic metabolism in systemic sclerosis (Scleroderma; SSc) and highlight the most appropriate targets for anti-fibrotic therapies.

**Materials and methods:**

Dermal fibroblasts were isolated from five SSc patients and five healthy donors. Cells were cultured in medium with/without TGF-β1 and with/without ALK5, pan-PIM or ATM kinase inhibitors. Extracellular flux analyses were performed to evaluate glycolytic and mitochondrial respiratory function. The mitochondrial network in TMRM-stained cells was visualized by confocal laser-scanning microscopy, followed by semi-automatic analysis on the ImageJ platform. Protein expression of ECM and fibroblast components, glycolytic enzymes, subunits of the five OXPHOS complexes, and dynamin-related GTPases and receptors involved in mitochondrial fission/fusion were assessed by western blotting.

**Results:**

Enhanced mitochondrial respiration coupled to ATP production was observed in SSc fibroblasts at the expense of spare respiratory capacity. Although no difference was found in glycolysis when comparing SSc with healthy control fibroblasts, levels of phophofructokinase-1 isoform PFKM were significantly lower in SSc fibroblasts (*P*<0.05). Our results suggest that the number of respirasomes is decreased in the SSc mitochondria; however, the organelles formed a hyperfused network, which is thought to increase mitochondrial ATP production through complementation. The increased mitochondrial fusion correlated with a change in expression levels of regulators of mitochondrial morphology, including decreased levels of DRP1, increased levels of MIEF2 and changes in OPA1 isoform ratios. TGF-β1 treatment strongly stimulated glycolysis and mitochondrial respiration and induced the expression of fibrotic markers. The pan-PIM kinase inhibitor had no effect, whereas both ALK5 and ATM kinase inhibition abrogated TGF-β1-mediated fibroblast activation, and upregulation of glycolysis and respiration.

**Conclusions:**

Our data provide evidence for a novel mechanism(s) by which SSc fibroblasts exhibit altered metabolic programs and highlight changes in respiration and dysregulated mitochondrial morphology and function, which can be selectively targeted by small molecule kinase inhibitors.

## Introduction

Systemic sclerosis (scleroderma, SSc) is a rare immune-mediated inflammatory disease of unknown etiology (1, 2). One of the most characteristic pathological manifestations is the connective tissue fibrosis (3). This is especially marked in the diffuse cutaneous form of the disease, where overproduction of collagen and other extracellular matrix (ECM) proteins by connective tissue fibroblasts results in excessive ECM deposition. The fibroblasts are activated and differentiated into hyperproliferative myofibroblasts by growth factors and cytokines, such as transforming growth factor-β1 (TGF-β1). Progressive replacement of tissue architecture by the collagen-rich ECM results in a functional impairment of the affected organs. The fibrotic process is most prominent in the skin, lungs, gastrointestinal tract, heart, tendons and ligaments, and endocrine glands. Fibrotic damage to these organs accounts for much of the morbidity and mortality. Although clinical outcomes have improved, SSc is still considered incurable and difficult to treat (4).

There are two main biochemical pathways in the cell that generate metabolic energy in the form of ATP: glycolysis in the cytosol and oxidative phosphorylation in the mitochondria. Glycolysis converts glucose to pyruvate with the concomitant production of a small amount of ATP. Under aerobic conditions, pyruvate is imported into the mitochondria and further broken down in the tricarboxylic acid (TCA) cycle. Products of the TCA cycle (NADH, FADH_2_) are subsequently oxidized, and the free energy of the redox reactions is used to sustain a proton electrochemical gradient across the inner mitochondrial membrane, which is used to drive ATP synthesis (5). This process is called oxidative phosphorylation and is the main source of ATP in aerobic cells. It has been known for a long time that, unlike normal tissues, most cancers use glycolysis as main bioenergetic pathway to produce ATP, even in the presence of oxygen (6). This aerobic glycolysis is known as the Warburg effect and is thought to be important to increase anabolic metabolism (biosynthesis of building blocks) and resistance to apoptosis in hyperproliferative cancer cells (7, 8).

It is increasingly recognized that re-programming of cellular energy metabolism also occurs in fibrotic diseases (9). For instance, keloid “scar” fibroblasts, which share characteristics with fibroblasts from SSc patients, were reported to use glycolysis as their primary energy source (10). A switch to glycolysis was also found in lung tissue and myofibroblasts from patients with idiopathic pulmonary fibrosis (IPF) (11, 12). During early myofibroblast differentiation, the expression of the glycolytic enzymes hexokinase 2 (HK2), phosphofructokinase 1 and 6-phosphofructo-2-kinase/fructose-2,6-biphosphatase 3 (PFKFB3) were found to increase and remained high. Pharmacological inhibition of glycolysis, attenuated the profibrotic phenotype, suggesting that the increased glycolytic flux is essential for development of the fibrosis (12). In addition, alveolar type II cells in IPF patients were found to exhibit marked accumulation of dysfunctional mitochondria (13). Likewise, vascular pulmonary cells in patients with pulmonary arterial hypertension (PAH) showed mitochondrial abnormalities (14) and a shift towards glycolysis, which is likely to play a causal role in the deleterious vascular remodeling because drug-induced reversal of the metabolic shift prevented remodeling (15). Similarly, differentiation of cultured hepatic stellate cells into myofibroblasts was shown to induce glycolysis (16). Finally, in mouse unilateral ureter obstruction-induced renal fibrosis and TGF-β1-treated renal interstitial fibroblasts, high levels of glucose uptake, glycolytic enzymes and lactate production were observed. Pharmacological inhibition of pyruvate kinase type M2 (PKM2) phosphorylation at Tyr residue 105, which is known to suppress aerobic glycolysis, attenuated renal fibrosis and fibroblast activation (17).

As current treatments of SSc are limited and have substantial side effects, new insights into the pathological processes are needed to develop novel therapeutic approaches. We hypothesize that rewiring of the energy generating pathways contribute to the disease process of SSc to meet the increased demand of energy and anabolic metabolism for fibrogenesis. The aims of the current study were: (a) to investigate possible changes in energy metabolism in cell culture models of SSc, (b) to explore the molecular basis of these changes, and (c) to develop selective therapeutic strategies that target the disrupted metabolism and stop fibrosis.

## Material and methods

### Cell culturing

Primary dermal fibroblast cultures were established from skin explants of five early diffuse SSc patients (≤2 y from first non-Raynaud’s symptom) and five unrelated, healthy, age-matched control subjects (**Supplementary Table S1**) according to standard procedures (18). From the patients, we took paired skin samples from lesional and uninvolved tissue. Donors provided prior informed written consent. Ethical approval was obtained from the Research Ethics Committee (reference: 6398) of the NHS Health Research Authority (NRES Committee London-Hampstead) in compliance with national legislation and the Declaration of Helsinki.

Cell passages at 3−7 were used for study. Cells were cultured in Dulbecco’s modified Eagle medium (DMEM) containing GlutaMAX and 25 mM glucose (Gibco, ThermoFisher Scientific), supplemented with 10% fetal bovine serum (FBS), 1 mM sodium pyruvate, 50 units/ml of penicillin and 50 μg/ml of streptomycin (culture medium) at 37°C in a humidified atmosphere of 5% CO_2_ in air. For the experiments with TGF-β1, cells were serum-starved in culture medium containing 0.1% FBS for 24 h before treatment with or without 2 ng/ml of TGF-β1 (R&D Systems) and with or without 10 μM SB431542 (Tocris Bioscience), 20 μM AZD1208 (Tocris Bioscience) or 5 μM KU55933 (Tocris Bioscience) for 24 h in fresh culture medium containing 0.1% FBS. Thousand-fold stock solutions were prepared in water (TGF-β1) or dimethyl sulfoxide (small molecule inhibitors) and stored at -80°C. The concentration of the inhibitors was informed by the literature and verified by viability assays using PrestoBlue Cell Viability Reagent (ThermoFisher Scientific), whereby only doses at which no toxic affects occurred were chosen.

### Western blot analysis

For the study of α-smooth muscle actin (α-SMA) and ECM proteins, cell cultures were washed twice with cold phosphate-buffered saline (PBS) followed by extraction in cold RIPA buffer (Merck, Sigma-Aldrich), supplemented with Roche cOmplete Protease Inhibitor Cocktail (Merck, Sigma-Aldrich). Cell monolayers were collected with a cell scraper, centrifuged for 10 min at 16,000 × *g*, 4°C, and supernatants were stored at -20°C for western blot analyses.

For the study of glycolytic pathway and mitochondrial proteins, cell cultures were washed with PBS and, subsequently, dislodged by trypsinization, collected by centrifugation, washed with PBS and extracted with 1% Triton X-100 in PBS, supplemented with protease inhibitors (1 μM phenylmethylsulfonyl fluoride, 1 μg/ml of pepstatin A and 1 μg/ml of leupeptin) and Phosphatase Inhibitor Cocktail 2 and 3 (Merck, Sigma-Aldrich). After 15 min on ice, samples were centrifuged for 10 min at 16,000 × *g*, 4°C, and the supernatants were stored at -80°C for western blot analyses.

Protein concentrations of the samples were determined with the Pierce BCA Protein Assay Kit (ThermoFisher Scientific) as detailed below. To prepare western blots, 10-μg samples in 1× Laemmli Sample Buffer (BioRad) and 1× NuPAGE Sample Reducing Agent (ThermoFisher Scientific) were resolved on 4−20% or 7.5% Criterion TGX Stain-Free Precast Gels (Biorad) alongside SeeBlue Plus2 Pre-stained Protein (ThermoFisher Scientific) or Precision Plus Protein (BioRad) standards and blotted onto Trans-Blot Turbo 0.2-μm PVDF membranes (BioRad) using a BioRad Trans-Blot Transfer System. Protein binding sites on the blots were saturated with 5% bovine serum albumin (BSA; for detection of phospho-proteins) or 10% skimmed milk powder (for detection of all other proteins) in PBS for 1 h, followed by a rinse with PBS, 0.3% Tween-20 and primary antibody incubation in PBS, 0.3% Tween-20, at 4°C, overnight. The primary antibodies are specified in **Supplementary Table S2**. Excess of primary antibodies was removed with three 10-min washes in PBS, 0.3% Tween-20, followed by a 1-h incubation with the appropriate horse radish peroxidase-conjugated secondary antibodies (Dako; P0160, P0447 and P0448) in PBS, 0.3% Tween-20, and another 3 washes. Blots were developed with Clarity Western ECL Substrate (BioRad). Capturing of the chemiluminescent signals was performed with a BioRad Chemidoc MP Imaging System. Signals were quantified with BioRad Image Lab 5.1 software. Afterwards, all blots were probed with an antibody against β-tubulin to verify loading. Antibody signals were normalized with the aid of the anti-β-tubulin signal and expressed relative to the mean value of the control samples.

### Extracellular flux analyses

Extracellular flux assays were performed on a Seahorse XFp platform (Agilent Technologies). To count the cells for seeding in the XFp cell culture microplates, they were dislodged by trypsinization, collected by centrifugation and resuspended in culture medium, followed by trypan blue exclusion cell counting on C-Chip Neubauer Improved Disposable Hemocytometer slides (NanoEnTek) in duplicate. Fibroblasts were seeded in wells of an XFp cell culture microplate at a density of 8,000 cells/well in 80 μl of culture medium and cultured for 24 h. In the TGF-β1 experiments, cells were serum-starved in the wells of the XFp cell culture microplate for a further 24 h prior to treatment with or without 2 ng/ml of TGF-β1 and with or without 10 μM SB431542, 20 μM AZD1208 or 5 μM KU55933 for 24 h in fresh culture medium containing 0.1% FBS.

To examine glycolytic function in glycolysis stress tests, the culture medium in the wells was replaced with 175 μl XFp Base medium (Agilent Technologies) pH 7.0 (NaOH), containing 2 mM l-glutamine, with or without TGF-β1 and with or without small molecule inhibitors. After 1 h of humidified incubation at 37°C, glycolysis was evaluated on the Seahorse platform. After three measurements under basal conditions, glucose was injected to a final concentration of 10 mM, followed by three measurements. Then, oligomycin A was injected to a final concentration of 1 μM, followed by three measurements. Last of all, 2-deoxy-d-glucose was injected to a final concentration 50 mM, followed by three measurements. Glucose was prepared as a 2.5 M stock solution in water and stored at room temperature, while oligomycin A (Merck, Sigma-Aldrich) was prepared as a 10 mM stock solution in ethanol and 2-deoxy-d-glucose (Merck, Sigma-Aldrich) was prepared as a 500 mM stock solution in XFp Base medium pH 7.0 and both stored at -20°C.

To examine mitochondrial respiratory function in mitostress tests, the culture medium in the wells was replaced with 175 μl XFp Base medium pH 7.0 (NaOH), containing 10 mM d-(+)-glucose, 1 mM sodium pyruvate and 2 mM l-glutamine, with or without TGF-β1 and with or without small molecule inhibitors. After 1 h of humidified incubation at 37°C, respiration was assessed on the Seahorse platform. After three measurements under basal conditions, oligomycin A was injected to a final concentration of 1 μM, followed by three measurements. Then, carbonyl cyanide 4-(trifluoromethoxy)phenylhydrazone (FCCP) was injected to a final concentration of 2 μM, followed by three measurements. To finish, rotenone and antimycin A were injected, both at a final concentration of 1 μM, followed by three measurements. Oligomycin A, FCCP, rotenone and antimycin A were purchased from Merck (Sigma-Aldrich). They were prepared as 10 mM stock solutions in ethanol and stored at -20°C.

On each XFp cell culture plate, two different cultures were compared in triplicate, e.g., fibroblasts from a control subject and fibroblasts from a SSc patient, or fibroblasts from a SSc patient treated with vehicle and fibroblasts from the same SSc patient treated with TGF-β1. All experiments were repeated independently 4 (**Figure 8**) or 6 (**Figure 2**) times by conducting two experiments per cell passage of two or three subsequent passages. Data were analyzed with Wave Desktop 2.6.1 software and a Microsoft Excel macro provided by Agilent Technologies. Results are expressed per cell number or amount of protein determined after the extracellular flux assay. Cell numbers in each well of the XFp cell culture plate were determined with the CyQUANT Cell Proliferation Assay Kit (ThermoFisher Scientific) by adding 220 μl of CyQUANT GR dye/lysis solution to the aspirated wells, followed by mixing through pipetting up-and-down. Then, 200 μl of the mixture was transferred to wells of a 96-well black plate with clear bottoms (Greiner). An empty row on the 96-well plate was filled with 200 μl of a serial dilution of 0−25,000 cells in CyQUANT GR dye/lysis solution. The fluorescent signals were recorded at 480 nm excitation and 520 nm emission with a Synergy HT plate reader. In the TGF-β1 experiments, the amount of protein in aspirated wells of the XFp cell culture plates were determined with the Pierce BCA Protein Assay Kit and a serial dilution of BSA. Standard curves for the number of cells *versus* the fluorescent signal or the concentration of BSA *versus* the absorption at 561 nm were constructed in Microsoft Excel. Equations of the standard curves were used to calculate the cell number or amount of protein per well.

### Citrate synthase assays

The Triton X-100 extracts of the cell cultures prepared for western blot analyses were also used to determine citrate synthase activity. Assays were carried out in quadruplicate as described (19).

### Mitochondrial network analysis

Cells were seeded at low density in 35-mm μ-dishes with a glass bottom (Ibidi). After 3−4 days of culturing, cells were rinsed with Hanks’ balanced salt solution (HBSS; ThermoFisher Scientific), followed by incubation in 1 ml of 25 nM tetramethylrhodamine methyl ester (TMRM; Invitrogen, ThermoFisher Scientific) in HBSS. After 15 min of incubation, red fluorescent images (7 z-stacks of 0.15 μm) were captured with a Nikon Eclipse Ti-E inverted confocal laser-scanning microscope, equipped with a ×60 objective. Imaging data were collected with NIS-Elements software (Nikon). Semi-automated analysis of mitochondrial networks in the cultured cells was performed with the MiNA macro toolset (20) on the FIJI distribution of the ImageJ platform (National Institutes of Health). All 15 different fibroblast cultures were analyzed; five cultures derived from control subjects, five cultures derived from uninvolved skin of SSc patients, and five cultures derived from involved skin of the same SSc patients. Six to 12 randomly chosen microscopic fields were studied of each culture with 1−10 cells per field. In total 17−58 cells of each culture were studied.

### Statistical analyses

Graphs and statistical analyses were executed with GraphPad Prism software. Data are presented as mean ± standard deviation. As the sample size was too small to confirm normal distribution, we used non-parametric Kruskal-Wallis tests to examine statistical significance. Mann-Whitney tests were used for pairwise comparisons. Statistical significance levels were set to *P*<0.05 with Bonferroni correction for multiple pairwise comparisons.

## Results

### Expression levels of fibrotic markers

**Figure 1:**
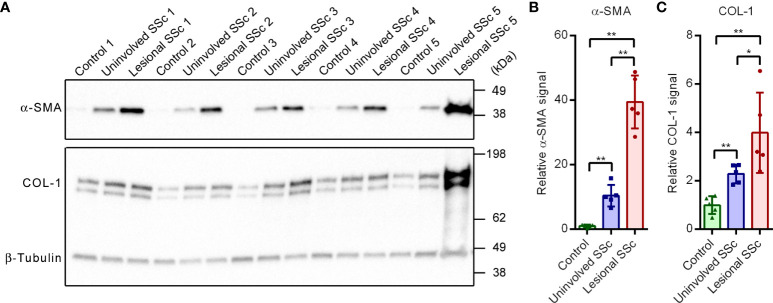
The fibrotic markers α-SMA and COL-1 are overexpressed in SSc fibroblasts. **(A)** Western blots with samples from five control, and five paired uninvolved and lesional SSc fibroblast cultures probed with antibodies against α-SMA, COL-1 and β-tubulin. Migration of protein standards is indicated. **(B, C)** Mean α-SMA and COL-1 signals, normalized for the β-tubulin signal and expressed relative to the mean value of the control samples. Data points show relative values of the individual samples. Error bars indicate standard deviations. Asterisks denote statistically significant differences (*P<0.05, **P<0.01).

In this study, we compared paired fibroblasts cultures derived from uninvolved and lesional skin of five SSc patients with dermal fibroblast cultures derived from five control subjects. First, we investigated the expression levels of two markers of fibrosis, α-smooth muscle actin (α-SMA) and collagen type 1 α1 chain (COL-1) on western blots (**Figure 1A**). Compared to the control samples, α-SMA and COL-1 were clearly overexpressed in the uninvolved SSc samples. In the lesional SSc samples, the expression levels of α-SMA and COL-1 were even higher (**Figures 1B, C**). These results confirm the fibrotic phenotype of the SSc fibroblasts.

### Glycolysis and mitochondrial respiration

**Figure 2:**
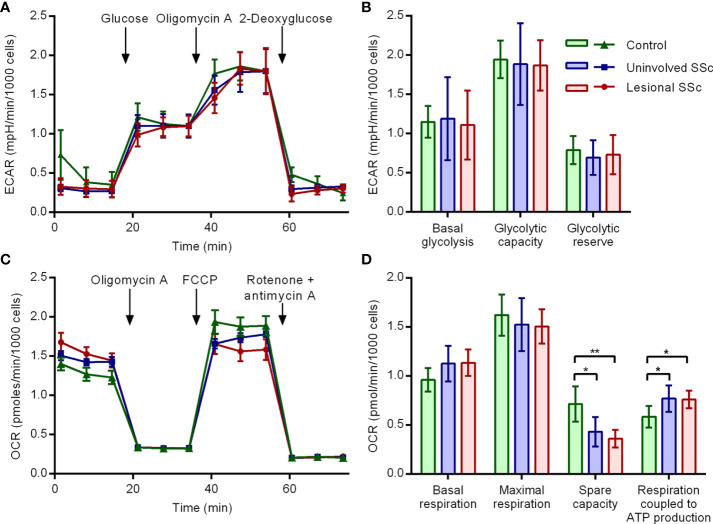
Mitochondrial OXPHOS is increased in SSc fibroblasts but glycolysis is unaffected. **(A)** Extracellular acidification rate (ECAR) profiles in representative glycolysis stress tests of a control, an uninvolved SSc and a corresponding lesional SSc fibroblast culture. Glucose, oligomycin A and 2-deoxyglucose were sequentially added to dissect glycolytic function. Error bars indicate standard deviation of technical triplicates. **(B)** Mean basal glycolysis, glycolytic capacity and glycolytic reserve of five control and five paired uninvolved and lesional SSc fibroblast cultures. Error bars indicate standard deviation. **(C)** Oxygen consumption rate (OCR) profiles in representative mitostress tests of a control, an uninvolved SSc and a corresponding lesional SSc fibroblast culture. Oligomycin A, FCCP and rotenone + antimycin A were sequentially added to dissect mitochondrial respiratory function. Error bars indicate standard deviation of technical triplicates. **(D)** Mean basal respiration, maximal respiration, spare respiratory capacity and respiration coupled to ATP production of five control and five paired uninvolved and lesional SSc fibroblast cultures. Error bars indicate standard deviation. Asterisks denote statistically significant differences (*P<0.05, **P<0.01).

To investigate whether energy metabolism in SSc fibroblasts is altered, we evaluated glycolysis and mitochondrial respiration with a Seahorse extracellular flux analyzer. We first determined the extracellular acidification rates (ECAR) as measure of glycolytic flux in glycolysis stress tests (**Figure 2A**). The experiments were repeated independently six times for each culture. The compiled data of the five pairs of SSc fibroblast cultures and five control cultures showed no differences in basal glycolysis, glycolytic capacity and glycolytic reserve (**Figure 2B**). Next, we determined the oxygen consumption rates (OCR) as measure of cellular respiration in mitostress tests (**Figure 2C**). Again, the experiments were repeated independently six times for each culture. The combined data for all 15 cultures revealed no differences in basal respiration and maximal respiration but indicated that the spare respiratory capacity was decreased in SSc fibroblasts, while the respiration coupled to ATP production was increased in SSc fibroblasts compared to the controls. Thus, the extracellular flux assays revealed a shift in mitochondrial respiration towards increased oxidative phosphorylation in SSc fibroblasts, while glycolytic flux did not change.

### Protein expression levels of enzymes of the glycolytic pathway and lactate secretion

**Figure 3:**
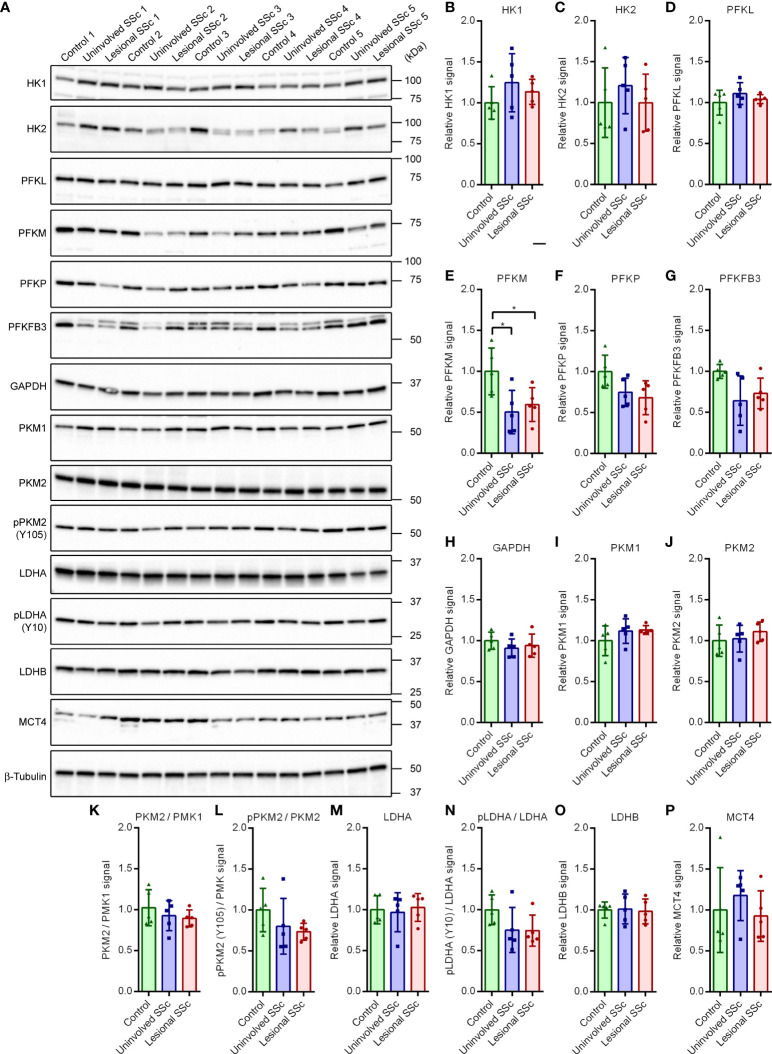
Muscle-type phosphofructokinase (PFKM) protein expression is decreased in SSc fibroblasts but the expression of other key enzymes of the glycolytic pathway and lactate secretion are unaffected. **(A)** Western blots with samples from five control, and five paired uninvolved and lesional SSc fibroblast cultures probed with antibodies against enzymes of the glycolytic pathway and lactate secretion as indicated. Blots were reprobed with an antibody against β-tubulin to confirm even loading. Migration of protein standards is shown. **(B−P)** Mean signals of the indicated proteins or protein ratios, normalized for the β-tubulin signal and expressed relative to the mean value of the control samples. Data points show relative values of the individual samples. Error bars indicate standard deviations. Asterisks denote statistically significant differences (*P<0.05).

Although the glycolytic stress tests did not suggest changes in glycolytic flux in the SSc fibroblasts, we investigated the glycolytic pathway and lactate secretion in more detail by looking at protein expression levels of key enzymes on western blots (**Figure 3A**). Hexokinase catalyzes the first step of glycolysis, the phosphorylation of glucose to glucose 6-phosphate (**Supplementary Figure S1**). The two most abundantly expressed isoenzymes HK1 and HK2 are critical for maintaining an elevated rate of glycolysis in cancer cells (21). Western blot analyses revealed no differences in HK1 and HK2 expression levels between control and SSc fibroblasts (**Figures 3B, C**).

Phosphofructokinase 1 catalyzes the phosphorylation of fructose 6-phosphate to fructose 1,6-bisphosphate in the rate-limiting, third step of glycolysis (**Supplementary Figure S1**). The active tetrameric enzyme is comprised of different combinations of PFKL, PFKM and PFKP subunit isoforms (22, 23). The western blots showed that levels of PFKL and PFKP were similar in control and SSc fibroblasts, but levels of PFKM were significantly lower in SSc samples compared to controls (**Figures 3D−F**).

The bifunctional 6-phosphofructo-2-kinase/fructose-2,6-bisphosphatase (PFKFB) catalyzes the synthesis and degradation of fructose 2,6-bisphosphate, thereby regulating its steady-state level (24). Fructose 2,6-bisphosphate is a positive allosteric regulator of phosphofructokinase 1 (**Supplementary Figure S1**). Four different isoforms of PFKFB have been identified. Isoform PFKFB3, which is highly expressed in most cancers, is inducible by hypoxia and promotes glycolysis under hypoxic conditions (25, 26). Our western blots did not show significant differences of PFKFB3 levels between control and SSc fibroblasts (**Figure 3G**).

Glyceraldehyde-3-phosphate dehydrogenase (GAPDH) converts glyceraldehyde-3-phosphate to 1,3 bisphosphoglycerate during glycolysis (**Supplementary Figure S1**). GAPDH is considered to be a constitutively expressed housekeeping protein (27). In agreement with this, we found very similar GAPDH levels in the control and SSc samples on a western blot (**Figure 3H**).

Pyruvate kinase catalyzes the conversion of phosphoenolpyruvate to pyruvate in the terminal step of glycolysis (**Supplementary Figure S1**). Isoform PKM1 is expressed in most adult tissues, whereas isoform PKM2 is expressed during embryonic development and is the dominant isoform in tumors (28). PKM2 can switch from a dimeric, inactive to a tetrameric, active form. Formation of tetrameric PKM2 is inhibited by phosphorylation at Tyr residue 105 (pPKM2 (Y105)). Phosphorylation promotes aerobic glycolysis (29). Moreover, in response to glucose restriction, PKM2 translocates to the nucleus and acts as a transcriptional co-activator or protein kinase in cancer stem cells, modulating the metabolic flux (30, 31). Our western blots showed no differences in the expression levels of PKM1 and PKM2, the ratio of PKM2 *versus* PKM1, or the degree of phosphorylation of PKM2 between control and SSc samples (**Figures 3I−L**).

Lactate dehydrogenase catalyzes the reversible conversion of pyruvate and NADH to lactate and NAD^+^. Isoform LDHA possesses a higher affinity for pyruvate and preferentially converts pyruvate and NADH to lactate and NAD^+^. In contrast, isoform LDHB has a higher affinity for lactate and preferentially converts lactate and NAD^+^ to pyruvate and NADH (**Supplementary Figure S1**). LDHA phosphorylated at residue Tyr10 (pLDHA (Y10)) is found in a variety of human cancer cells. Phosphorylation enhances LDHA activity to promote aerobic glycolysis (32). On western blots we found similar expression levels of LDHA and LDHB, and the degree of phosphorylation of LDHA (**Figures 3M−O**).

Monocarboxylate transporters transfer lactate, pyruvate and other monocarboxylates across the plasma membrane. MCT4 facilitates the release of lactate from the cell (**Supplementary Figure S1**). Its expression is promoted by catabolic transcription factors, such as nuclear factor κ-light-chain-enhancer of activated B cells (NF-κB) (33). Expression levels of MCT4 varied in the different cultures, but we did not detect significant differences between control and SSc fibroblasts on western blots (**Figure 3P**). Taken together, our western blot analysis of key enzymes of the glycolysis and lactate secretion revealed a decrease of PFKM protein expression levels in SSc fibroblasts compared to controls, but expression levels of other enzymes appeared unaffected.

### Expression levels of subunits of the oxidative phosphorylation enzyme complexes

**Figure 4:**
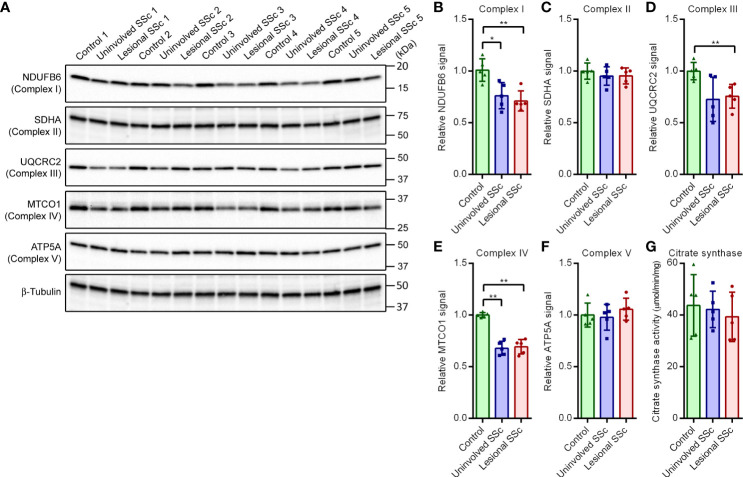
Subunits of OXPHOS enzyme complexes I, III and IV show decreased expression in SSc fibroblasts but subunits of OXPHOS enzyme complexes II and V, and citrate synthase activity are unaffected. **(A)** Western blots with samples from five control, and five paired uninvolved and lesional SSc fibroblast cultures probed with antibodies against subunits of the OXPHOS complexes as indicated. Blots were reprobed with an antibody against β-tubulin to confirm even loading. Migration of protein standards is shown. **(B−F)** Mean signals of the indicated proteins, normalized for the β-tubulin signal and expressed relative to the mean value of the control samples. Data points show relative values of the individual samples. **(G)** Mean citrate synthase activity of five control, and five paired uninvolved and lesional SSc fibroblast cultures. Data points show values of the individual samples. Error bars indicate standard deviations. Asterisks denote statistically significant differences (*P <0.05, **P<0.01).

The mitostress tests demonstrated increased oxidative phosphorylation in the SSc fibroblasts. To investigate this further, we determined the expression levels of representative subunits of each of the five oxidative phosphorylation enzyme complexes on western blots (**Figure 4A**). The blots indicated that expression levels of subunit NDUFB6 of Complex I were significantly decreased in SSc fibroblasts compared to controls (**Figure 4B**). Conversely, expression levels of the SDHA subunit of complex II were comparable in SSc and control fibroblasts (**Figure 4C**). Parallel to NDUFB6, however, the expression levels of the UQCRC2 subunit of Complex III and the MTCO1 subunit of Complex IV were decreased in SSc fibroblasts (**Figures 4D, E**). On the other hand, expression levels of the ATP5A subunit of Complex V showed no significant differences between SSc fibroblasts and controls (**Figure 4F**). Thus, our results suggest that the oxidative phosphorylation complexes I, III and IV that together form the respirasome supercomplex (34) are decreased in SSc fibroblasts, whereas levels of complexes II and V remain unchanged.

### Citrate synthase activity

Citrate synthase is an enzyme of the mitochondrial TCA cycle (**Supplementary Figure S1**). Citrate synthase is commonly used as biomarker for mitochondrial density in cells (35–37). Although citrate synthase activity showed some variation in the different fibroblast cultures, we did not observe significant differences between SSc and control cells (**Figure 4G**).

### Mitochondrial network morphology

**Figure 5:**
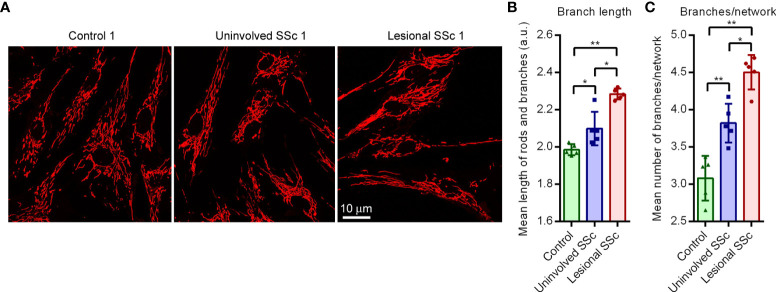
Cultured SSc fibroblasts have hyperfused mitochondrial networks. **(A)** Representative fluorescent micrographs of TMRM-stained control, uninvolved SSc and corresponding lesional SSc fibroblasts. **(B)** Mean length of mitochondrial rods and branches in arbitrary units (a.u.), and **(C)** mean number of branches per mitochondrial network in fibroblasts of five control and five paired uninvolved and lesional SSc cultures. Data point represent the mean length or the number of branches per network in 17−58 cells of the 15 individual cultures. Error bars indicate standard deviations. Asterisks denote statistically significant differences (*P <0.05, **P<0.01).

The mitochondrial network in cells is continuously re-modelled through fission and fusion events to adapt to changing physiological conditions (38). Mitochondrial fission generates new organelles and contributes to quality control by facilitating purging of damaged organelles *via* mitophagy. Mitochondrial fusion helps to mitigate stress through complementation of damaged mitochondria and is thought to increase mitochondrial ATP production. We investigated the mitochondrial network morphology in the SSc and control fibroblast cultures through live cell staining with the mitochondrial selective dye TMRM (**Figure 5A**). Analyses of the images revealed that there was no significant difference between the mitochondrial footprint per cell of SSc and control cultures; however, the mean length of the mitochondrial rods and branches, and the mean number of branches per mitochondrial network were increased in uninvolved SSc fibroblasts compared to controls. In the lesional SSc fibroblasts, these two parameters were still further increased (**Figures 5B, C**). These findings indicate that SSc fibroblasts have a hyperfused mitochondrial network.

### Expression levels of proteins involved in mitochondrial fission and fusion

**Figure 6:**
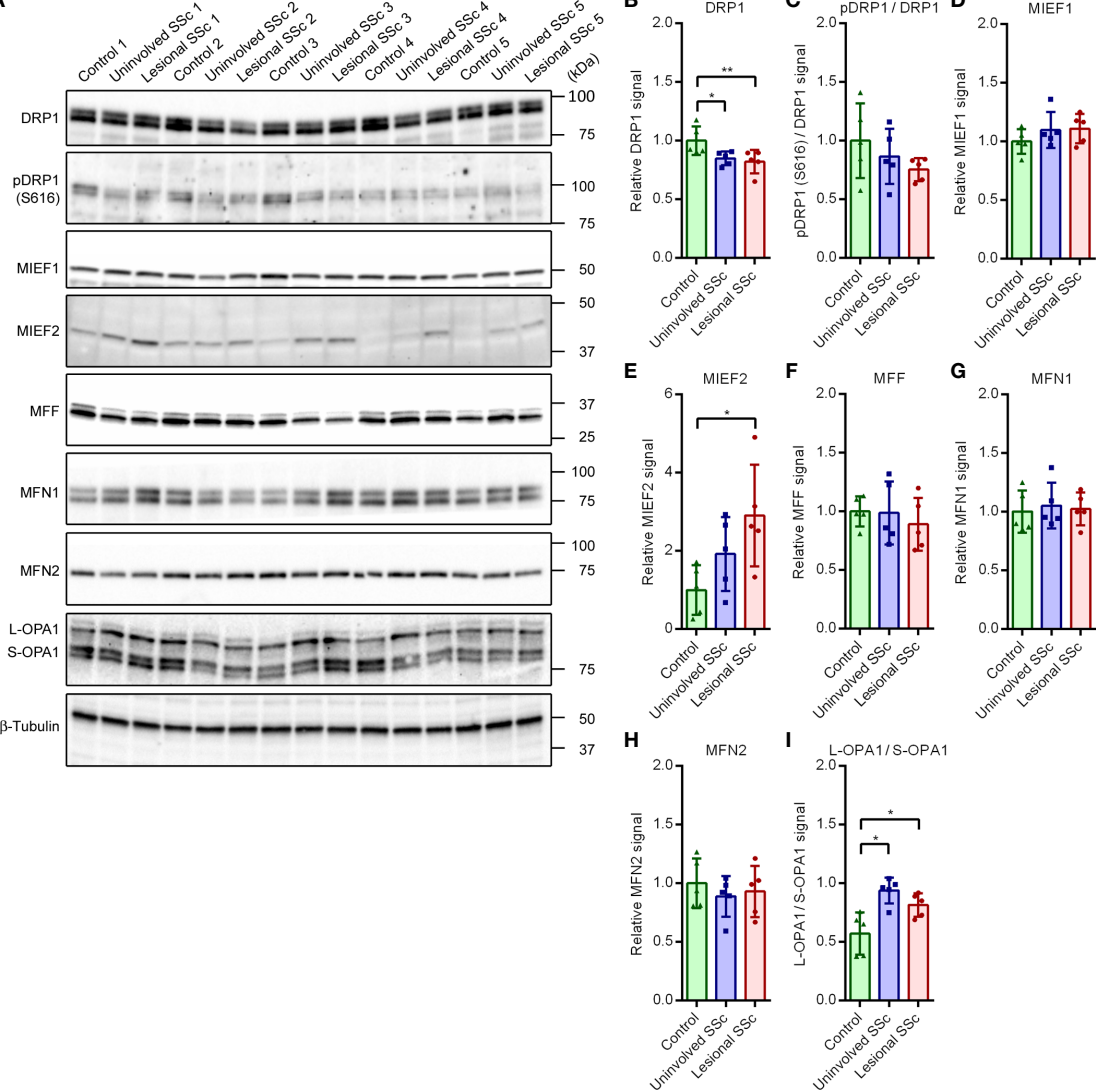
Expression of proteins engaged in mitochondrial fission and fusion shift in SSc fibroblasts to promote fusion. **(A)** Western blots with samples from five control, and five paired uninvolved and lesional SSc fibroblast cultures probed with antibodies against proteins involved in mitochondrial fission and fusion as indicated. Blots were reprobed with an antibody against β-tubulin to confirm even loading. Migration of protein standards is shown. **(B−I)** Mean signals of the indicated proteins, normalized for the β-tubulin signal and expressed relative to the mean value of the control samples or given as a ratio. Data points show relative values of the individual samples. Error bars indicate standard deviations. Asterisks denote statistically significant differences (*P<0.05, **P<0.01).

Mitochondrial network morphology is governed by the balance of mitochondrial fission and fusion events (39). To investigate how this balance is changed in SSc fibroblasts, we determined the expression levels of dynamin-related GTPases and adaptors/receptors that mediate mitochondrial modelling (**Supplementary Figure S1**) by western blot analyses (**Figure 6A**). The GTPase DRP1 is a mainly cytosolic protein that plays a central role in mitochondrial fission. Western blot analysis revealed that the expression levels of DRP1 were slightly, but statistically significant, lower in SSc fibroblasts compared to controls (**Figure 6B**). Phosphorylation of DRP1 at residue Ser616 (pDRP (S616)) promotes mitochondrial fission (40), whereas phosphorylation of DRP1 at residue Ser637 (pDRP (S637)) promotes mitochondrial fusion (41, 42). Our western blots showed that the ratios of pDRP (S616) *versus* total DRP1 were similar in the control and SSc samples (**Figure 6C**). We did not detect pDRP1 (S637) on western blots, even after prolonged exposure (**Supplementary Figure S2**). The recruitment of cytosolic DRP1 to mitochondria is a key step in mitochondrial fission. MFF, and the paralogues MIEF1 (also known as MiD51 or SMCR7L) and MIEF2 (also known as MiD49 or SMCR7) are the receptor and adaptor proteins for DRP1 recruitment to the mitochondrial surface (43, 44). We found comparable expression levels of MIEF1 and MFF in control and SSc samples; however, expression levels of MIEF2 were significantly higher in lesional SSc fibroblasts than in control fibroblasts (**Figures 6D−F**).

Fusion of the inner mitochondrial membrane is mediated by the GTPase OPA1, whereas fusion of the outer mitochondrial membrane is mediated by the GTPase paralogues MFN1 and MFN2 (39). Processing of so-called long OPA1 (L-OPA1) by multiple proteases generates short OPA1 (S-OPA1) forms (45). Fusion of the inner mitochondrial membrane depends on the equilibrium between L-OPA1 and S-OPA1. The western blots showed similar levels of MFN1 and MFN2 in control and SSc fibroblasts (**Figures 6G, H**). On the other hand, the ratios of L-OPA1 *versus* S-OPA1 were higher in the SSc samples than in the controls (**Figure 6I**). This may favor mitochondrial fusion.

### Expression levels of fibrotic markers in TGF-β1-activated fibroblasts treated with kinase inhibitors

When we compared the energy metabolism of SSc and healthy control fibroblasts, we observed no difference in glycolysis but noticed a small increase in respiration coupled to ATP production (**Figure 2**). To explore whether further induction of the fibrotic phenotype of SSc fibroblasts leads to additional increases in mitochondrial respiration and, possibly, glycolysis, cultures were treated with TGF-β1. This cytokine is a well-known activator of fibrotic genes and has long been implicated in the pathogenesis of SSc (46). In addition, we tested whether three small molecule kinase inhibitors, SB431542, AZD1208 and KU55933, were able to reverse the effects of TGF-β1 treatment. SB431542 is a specific inhibitor of the TGF-β1 activin receptor-like kinase (ALK)-4, -5 and -7 (47). AZD1208 is a specific inhibitor of Proviral Integration site for Moloney murine leukemia virus (PIM) kinase-1, -2 and -3 (48). KU55933 is a selective Ataxia-Telangiectasia Mutated (ATM) kinase inhibitor (49). These inhibitors are known to suppress matrix production, modulate cell proliferation and regulate differentiation programs (50–52).

**Figure 7:**
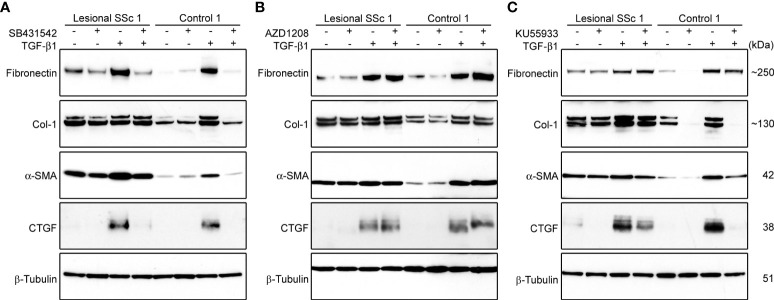
The kinase inhibitors SB431542 and KU55933, but not AZD1208, prevent TGF-β1-induced overexpression of α-SMA and ECM proteins in lesional SSc and control fibroblasts. **(A−C)** Western blots with samples from a lesional SSc and a control fibroblast culture treated vehicle or 2 ng/ml of TGF-β1 and/or 10 μM SB431542, 20 μM AZD1208 or 5 μM KU55933, and probed with antibodies against α-SMA or ECM proteins as indicated. Blots were reprobed with an antibody against β-tubulin to verify even loading.

First, we investigated the effect of TGF-β1 and the three kinase inhibitors on the expression levels of the fibrotic markers fibronectin, Col-1, α-SMA, and Connective Tissue Growth Factor (CTGF) in lesional SSC and control fibroblast cultures. Western blots showed that TGF-β1 treatment markedly induced the expression levels of the four fibrotic markers in both cell types (**Figure 7**). Treatment with SB431542 completely prevented the increase in expression of the fibrotic markers in TGF-β1-treated cells (**Figure 7A**; **Supplementary Figures S3A–D**). AZD1208 treatment, in contrast, had no effect on the expression levels of the fibrotic markers (**Figure 7B**; **Supplementary Figures S3E–H**), whereas KU55933 prevented the TGF-β1-induced expression of COL-1, α-SMA and CTGF in control and lesional fibroblasts (**Figure 7C**; **Supplementary Figures S3I–L**).

### Glycolysis and mitochondrial respiration in TGF-β1-activated lesional SSc fibroblasts treated with kinase inhibitors

**Figure 8:**
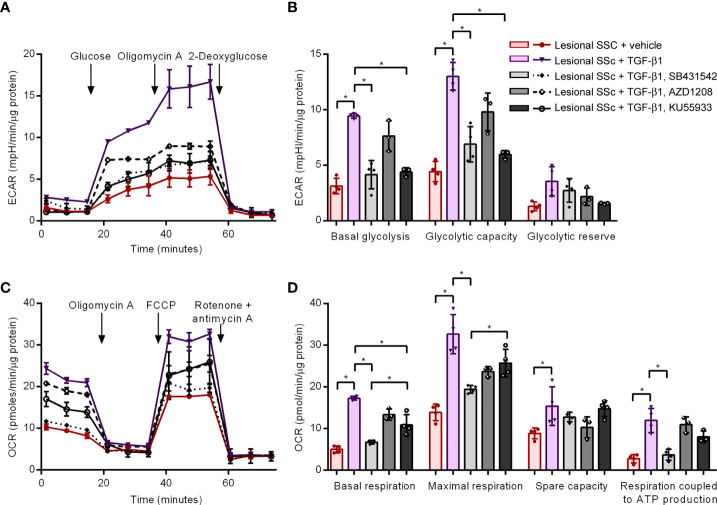
The kinase inhibitors SB431542 and KU55933, but not AZD1208, prevent TGF-β1-induced increases in glycolysis and mitochondrial respiration in lesional SSc fibroblasts. **(A)** Extracellular acidification rates (ECAR) in representative glycolysis stress tests of a lesional SSc fibroblast culture treated vehicle or 2 ng/ml of TGF-β1 with or without 10 μM SB431542, 20 μM AZD1208 or 5 μM KU55933. Glucose, oligomycin A and 2-deoxyglucose were sequentially added to delineate glycolytic function. Error bars indicate standard deviation of technical triplicates. **(B)** Mean basal glycolysis, glycolytic capacity and glycolytic reserve of four lesional SSc fibroblast cultures treated vehicle or 2 ng/ml of TGF-β1 with or without 10 μM SB431542, 20 μM AZD1208 or 5 μM KU55933. Error bars indicate standard deviation. **(C)** Oxygen consumption rates (OCR) in representative mitostress tests of a lesional SSc fibroblast culture treated vehicle or 2 ng/ml of TGF-β1 with or without 10 μM SB431542, 20 μM AZD1208 or 5 μM KU55933. Oligomycin A, FCCP and rotenone + antimycin A were sequentially added to delineate mitochondrial respiratory function. Error bars indicate standard deviation of technical triplicates. **(D)** Mean basal respiration, maximal respiration, spare respiratory capacity and respiration coupled to ATP production of four lesional SSc fibroblast cultures treated vehicle or 2 ng/ml of TGF-β1 with or without 10 μM SB431542, 20 μM AZD1208 or 5 μM KU55933. Error bars indicate standard deviation. Asterisks denote statistically significant differences (*P<0.05).

Finally, we studied of effects of TGF-β1 activation with or without SB431542, AZD1208 or KU55933 treatment on energy metabolism in lesional SSc fibroblast cultures. We first performed glycolytic stress tests on the Seahorse platform (**Figure 8A**). The combined results of four different lesional SSc cultures demonstrated that TGF-β1 activation tripled the basal glycolysis as well as the glycolytic capacity (**Figure 8B**). SB431542 and KU55933 treatment counteracted the glycolytic stimulation of TGF-β1 but AZD1208 treatment had no significant effect. We then carried out mitostress tests to assess mitochondrial respiration (**Figure 8C**). The compiled data of four different lesional SSc cultures indicated that basal respiration and respiration coupled to ATP production quadrupled after TGF-β1 activation, while maximal respiration more than doubled and spare respiratory capacity increased by about half (**Figure 8D**). SB431542 treatment prevented the stimulatory effects of TGF-β1 on basal and maximal respiration, and respiration coupled to ATP production. AZD1208 treatment had no significant effect on TGF-β1-stimulated respiration. KU55933 treatment significantly reduced the TGF-β1-stimulated basal respiration but not as potent as SB431542.

## Discussion

The objective of this study was to investigate the remodeling of energy metabolism in cell culture models of SSc in order to develop therapeutic strategies that target the disrupted metabolism and reverse the fibrotic phenotype. Although metabolic remodeling has been documented in other fibrotic diseases, studies of the role of energy metabolism in SSc are limited (53, 54). First, we compared paired fibroblasts cultures derived from uninvolved and lesional skin of five SSc patients with dermal fibroblast cultures from five control subjects. Western blot analyses for the fibrotic markers α-SMA and COL-1 confirmed the fibrotic phenotype of SSc fibroblasts. Glycolytic stress tests did not reveal differences in glycolysis between SSC and control cultures. It is of course possible that our experiments are underpowered and that a larger number of patient and control samples would reveal a difference. The mitostress tests, however, exposed a small but significant increase in mitochondrial respiration coupled to ATP production at the expense of the spare respiratory capacity. These results suggest that SSc fibroblasts boost their oxidative phosphorylation to meet the increased bioenergetic demand of enhanced ECM protein synthesis and proliferation.

Assessment of the expression levels of key, rate-limiting enzymes of the glycolytic pathway and lactate secretion on western blots showed that levels of the phosphofructokinase 1 isoform PFKM were significantly lower in SSc fibroblasts compared to control fibroblasts, while levels of the isoforms PFKL and PFKP were unaffected. Of the three isoforms, PFKM has the highest affinities for fructose 6-phosphate and ATP. In addition, PFKM is the least sensitive to fructose 2,6-bisphosphate allosteric modulation and ATP inhibition (23). In other words, PFKM is the most active enzyme but is less adaptable than PFKL and PFKP. Therefore, the lower PFKM expression suggests that glycolytic regulation may be of greater importance than activity for SSc fibroblast.

We also determined the expression levels of representative subunits of each of the five oxidative phosphorylation enzyme complexes by western blotting. Our results indicated that subunits of the oxidative phosphorylation complexes I, III and IV are decreased by ~25% in SSc fibroblasts compared to control samples, whereas levels of complexes II and V remain unchanged. The mitochondrial footprint and the activity of the TCA cycle enzyme citrate synthase in SSc fibroblasts corresponded to that of controls, suggesting that the mitochondrial mass in the cells is unaffected. Complexes I, III and IV form a supercomplex called the respirasome (34). When taken together, our results suggest that the number of respirasomes decrease in mitochondria of SSc fibroblasts. This is an unexpected finding because basal respiration and respiratory capacity did not change in SSc fibroblasts. Possibly, post-transcriptional modifications of the respirasome modulate its activity to increase coupling to ATP production. Mitochondrial morphology is controlled by energy metabolism. Live cell staining of the mitochondrial network demonstrated that SSc fibroblasts have a hyperfused network. Mitochondrial fusion is known to be driven by bioenergetic demand and stress (38). Fusion of mitochondria is thought to increase mitochondrial ATP production through complementation. Thus, the mitochondrial hyperfusion in SSc fibroblasts may promote respiration coupled to ATP production, reflecting the increased bioenergetic demand of the cells.

When we investigated the molecular basis of mitochondrial hyperfusion in SSc fibroblasts by western blot analyses of proteins involved in mitochondrial modelling, we found that the fission promoting protein DRP1 was decreased in SSc fibroblasts compared to controls; however, we did not find changes in the level of post-transcriptional phosphorylation of DRP1. As the phosphorylation determines the activity of DRP1 (55), the decreased levels of total DRP1 in SSc fibroblasts do not explain the mitochondrial hyperfusion in these cells. The western blots also revealed that MIEF2 was increased in SSc fibroblasts. MIEF2 acts as an adaptor of DRP1 and its receptor MFF, linking DRP1 with MFF on the mitochondrial surface (44). The higher levels of MIEF2 may recruit inactive DRP1 to the mitochondrial surface resulting in mitochondrial elongation (56). Alternatively, the upregulation of MIEF2 may sequester DRP1 in DRP1-MIEF2-MFF as well as DRP1-MIEF2 complexes, inhibiting direct interaction between DRP1 and MFF and resulting in mitochondrial fusion (57). In addition, western blot analyses indicated that the ratio of L-OPA1 *versus* S-OPA1 were higher in SSc fibroblasts than in the control cells. The inner mitochondrial membrane embedded protein L-OPA1 is processed by multiple proteases to S-OPA proteins that have lost their membrane spanning N-terminus. Fusion of the inner membrane is determined by the balance between L-OPA1 and S-OPA1 (39). The observed shift towards L-OPA in SSc fibroblasts may favor mitochondrial fusion.

In the final experiments, we compared lesional SSc fibroblasts with their TGF-β1-induced myofibroblast derivatives to mimic a more advanced stage of fibrosis. Western blot analyses for the fibrotic markers CTGF, α-SMA, COL-1 and fibronectin demonstrated that TGF-β1 treatment induced the expression of all four fibrotic markers, confirming myofibroblast differentiation. To dissect the key signaling pathways in addition to determining the role of classical TGF-β1 signaling (*via* the kinase ALK5), we also explored the role of two other pathways regulated by PIM kinase (58) and ATM kinase (59) that have been shown to have a role in fibrosis. Co-treatment with the TGF-β1 receptor/ALK5 inhibitor SB431542 completely prevented the TGF-β1-induced expression of the fibrotic markers, whereas co-treatment with the pan-PIM kinase inhibitor AZ1208 was ineffective. Co-treatment with the ATM kinase inhibitor KU55933 prevented the TGF-β1-induced expression of COL-1, α-SMA and CTGF. Glycolytic and mitostress tests demonstrated that TGF-β1 robustly stimulated basal glycolysis and glycolytic capacity, as well as basal and maximal respiration, and respiration coupled to ATP production. TGF-β1-mediated stimulation of glycolysis and glutaminolysis, but not of mitochondrial respiration, was reported in normal human dermal fibroblasts by Henderson et al. (60). However, Bernard et al. (61, 62) and Bates et al. (63) reported a TGF-β1-mediated stimulation of glycolysis and mitochondrial respiration in normal human lung fibroblasts and human primary hepatic stellate cells, respectively. In addition, increased mitochondrial respiration and content has been documented during TGF-β1-mediated differentiation of NIH/3T3 mouse fibroblasts (64) and an upregulation of mitochondrial mass has been reported in TGF-β1-treated normal human lung fibroblasts (65). Thus, our results with SSc dermal fibroblasts are generally supported by findings in other laboratories. Interestingly, co-treatment with SB431542 and KU55933 neutralized the TGF-β1-mediated stimulation of glycolysis and mitochondrial respiration, but AZD1208 treatment had no significant effect. The differential impact of TGF-β1 signaling *via* the canonical Smad pathway, and the PIM and ATM kinase on fibroblast energy generation highlights additional pathways to those previously identified (mTOR/AMPK/p38) as potentially critical to fibroblast metabolism underpinning scarring and fibrosis (61, 66, 67). The interaction(s) between these pathways and the mechanisms involved leading to fibrogenesis are being actively studied and once fully elucidated will allow identification of new markers and crucial candidates for the management and treatment of tissue fibrosis.

## Data availability statement

The raw data supporting the conclusions of this article will be made available by the authors, without undue reservation.

## Ethics statement

This study was reviewed and approved by Research Ethics Committee (reference: 6398) of the NHS Health Research Authority (NRES Committee London-Hampstead). The patients/participants provided their written informed consent to participate in this study.

## Author contributions

Conception and design: DA, JA-H and J-WT. Provision of patients: VO and CD. Collection and preservation of cell cultures: XS. Collection and analysis of results: IC, HL (Xi’an), HL (UCL), VR and JWT. Drafting of manuscript and preparation of figures: J-WT. Editing of manuscript: MP, GX, JL-F, JA-H, DA and J-WT. All authors contributed to the article and approved the submitted version.

## Funding

IC was supported by the Conselho Nacional de Desenvolvimento Científico e Tecnológico and Ciência Sem Fronteiras, Brazil. HL (Xi’an) was a recipient of a University Graduate Students Studying Abroad awarded from the Chinese Scholarship Council. HL (UCL) was awarded a Wellcome Trust Biomedical Vacation Scholarship (code: 213295/Z/18/Z). DA obtained funding from Versus Arthritis (formerly Arthritis Research UK; codes: 21810 and 19427), the Royal Free Charity (Fund 97), Scleroderma and Raynaud’s UK, and The Rosetrees Trust (code: M96-F1/F2). J-WT obtained funding from the Royal Free Charity (Fund 42).

## Acknowledgments

We acknowledge the support from Ms Korsa Khan and Ms Bahja Ahmed Abdi for laboratory management, and Dr Ioannis Papaioannou for technical expertise.

## Conflict of interest

The authors declare that the research was conducted in the absence of any commercial or financial relationships that could be construed as a potential conflict of interest.

## Publisher’s note

All claims expressed in this article are solely those of the authors and do not necessarily represent those of their affiliated organizations, or those of the publisher, the editors and the reviewers. Any product that may be evaluated in this article, or claim that may be made by its manufacturer, is not guaranteed or endorsed by the publisher.
