# Supplementary material for: Exploring metabolism in scleroderma reveals opportunities for pharmacological intervention for therapy in fibrosis
Source: Front Immunol. 2022 Oct 11;13:1004949. doi: 10.3389/fimmu.2022.1004949 (PMC9592691; doi:10.3389/fimmu.2022.1004949)
Supplement: Supplementary file 1 [file Table_1.pdf]

Supplementary Table S1 | Age, sex and clinical details of patients and control subjects.

Dermal fibroblast culture	Age at biopsy (y)	Sex	Auto-antibody status	Symptoms	Modified Rodnan skin score at biopsy	Medication
P1	60	F	Scl70 +	dcSSc<br>Lung fibrosis	32	Cyclophosphamide<br>MFF
P2	37	M	ANA +<br>ENA -	dcSSc<br>Renal crisis<br>Lung fibrosis	23	MMF
P3	60	F	ARA +<br>(pol3+)	dcSSc	26	MMF
P4	49	F	ARA +<br>(pol3+)	dcSSc<br>Lung fibrosis<br>Myositis	32	MMF
P5	55	F	ARA +<br>(pol3+)	dcSSc	28	MMF
C1	56	M	-	-	-	-
C2	44	F	-	-	-	-
C3	57	F	-	-	-	-
C4	43	F	-	-	-	-
C5	55	F	-	-	-	-

Abbreviations: ANA, anti-nuclear antibodies; ARA, anti-RNA polymerase antibodies; ENA, extractable nuclear antigen antibodies; dcSSC, diffuse cutaneous systemic sclerosis; MMF, Mycophenolate mofetil
