# Supplementary material for: Exploring metabolism in scleroderma reveals opportunities for pharmacological intervention for therapy in fibrosis
Source: Front Immunol. 2022 Oct 11;13:1004949. doi: 10.3389/fimmu.2022.1004949 (PMC9592691; doi:10.3389/fimmu.2022.1004949)
Supplement: Supplementary file 2 [file Table_2.pdf]

**Supplementary Table S2 |** Primary antibodies used for western blot analyses

| Target | Supplier | Catalogue number | Dilution |
|---|---|---|---|
| ATP5A | Abcam | ab14748 | $6,000^{-1}$ |
| COL-1 | Millipore | AB758 | $3,000^{-1}$ |
| CTGF | Abcam | ab6992 | $1,000^{-1}$ |
| DRP1 | BD Transduction Laboratories | 611113 | $1,000^{-1}$ |
| Fibronectin | Calbiochem | | $1,000^{-1}$ |
| GAPDH | Abcam | ab8245 | $120,000^{-1}$ |
| HK1 | Cell Signaling Technology | 2024 | $1,000^{-1}$ |
| HK2 | Cell Signaling Technology | 2106 | $1,000^{-1}$ |
| LDHA | Proteintech | 19987-1-AP | $3,000^{-1}$ |
| LDHB | Proteintech | 14824-1-AP | $3,000^{-1}$ |
| MCT4 | Proteintech | 22787-1-AP | $1,500^{-1}$ |
| MFF | Cell Signaling Technology | 86668 | $1,000^{-1}$ |
| MFN1 | Abcam | ab57602 | $1,000^{-1}$ |
| MFN2 | Abcam | ab56889 | $1,000^{-1}$ |
| MIEF1 | Proteintech | 20164-1-AP | $2,000^{-1}$ |
| MIEF2 | Proteintech | 16413-1-AP | $500^{-1}$ |
| MTCO1 | Abcam | ab14705 | $3,000^{-1}$ |
| NDUFB6 | Abcam | ab110244 | $3,000^{-1}$ |
| OPA1 | BD Transduction Laboratories | 612607 | $1,000^{-1}$ |
| pDRP1 (S616) | Cell Signaling Technology | 3455 | $750^{-1}$ |
| pDRP1 (S637) | Abcam | ab193216 | $500^{-1}$ |
| PFKFB3 | Cell Signaling Technology | 13123 | $1,500^{-1}$ |
| PFKL | Abcam | ab97443 | $1,500^{-1}$ |
| PFKM | Proteintech | 55028-1-AP | $1,500^{-1}$ |
| PFKP | Cell Signaling Technology | 8164 | $1,500^{-1}$ |
| PKM1 | Cell Signaling Technology | 7067 | $1,500^{-1}$ |
| PKM2 | Cell Signaling Technology | 4053 | $1,500^{-1}$ |
| pLDHA (Y10) | Cell Signaling Technology | 8176 | $750^{-1}$ |
| pPKM2 (Y105) | Cell Signaling Technology | 3827 | $1,000^{-1}$ |
| SDHA | Abcam | ab14715 | $3,000^{-1}$ |
| UQCRC2 | Abcam | ab14745 | $3,000^{-1}$ |
| α-SMA | Agilent Dako | M085101-2 | $3,000^{-1}$ |
| β-Tubulin | Abcam | ab6046 | $12,000^{-1}$ |
