# Supplementary material for: Exploring metabolism in scleroderma reveals opportunities for pharmacological intervention for therapy in fibrosis
Source: Front Immunol. 2022 Oct 11;13:1004949. doi: 10.3389/fimmu.2022.1004949 (PMC9592691; doi:10.3389/fimmu.2022.1004949)
Supplement: Supplementary file 3 [file Image_1.pdf]

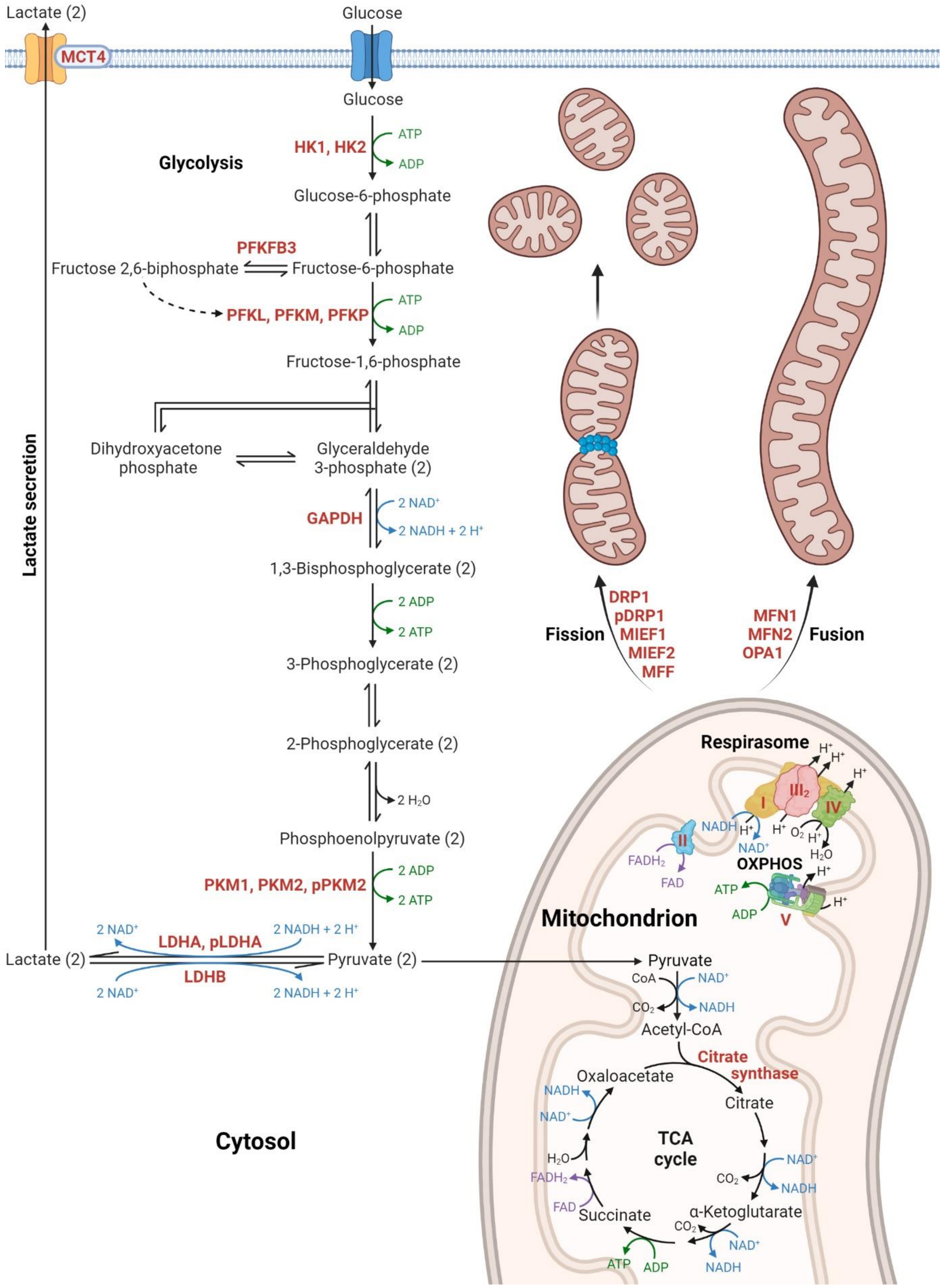

Supplementary Figure S1 | Aspects of energy metabolism investigated in SSc fibroblast cultures. In this study, we determined the glycolytic and mitochondrial respiratory flux with glycolysis and mitostress tests, respectively. Western blot analysis was used to assess the levels of key enzymes of the glycolysis and lactate secretion pathways, including hexokinase 1 and 2 (HK1, HK2), 6-phosphofructo-2-kinase/fructose-2,6-bisphosphatase 3 (PFKFB3),

phosphofructokinase 1 isoforms PFKL, PFKM and PFKP, glyceraldehyde-3-phosphate dehydrogenase (GAPDH), pyruvate kinase 1 and 2 (PKM1, PKM2), phospho-PKM2 (pPKM2; Y105), lactate dehydrogenase A and B (LDHA, LDHB), phospho-LDHA (pLDHA; Y10), and the monocarboxylate transporter MCT4. The activity of the tricarboxylic acid (TCA) cycle enzyme citrate synthase was determined in biochemical assays. Western blot analysis was also used to measure the levels of essential subunits of the oxidative phosphorylation (OXPHOS) enzyme complexes I, II, III, IV and V. The mitochondrial network morphology was evaluated by confocal microscopy. Finally, western blot analysis was used to assess levels of proteins involved in mitochondrial fission and fusion, including dynamin-related protein 1 (DRP1), phospho-DRP1 (pDRP1; S616), mitochondrial elongation factor 1 and 2 (MIEF1, MIEF2), mitochondrial fission factor (MFF), mitofusin 1 and 2 (MFN1, MFN2) and optic atrophy 1 (OPA1). Figure was created in BioRender.com (Agreement number: IL242PYGNE).
