# Supplementary material for: Exploring metabolism in scleroderma reveals opportunities for pharmacological intervention for therapy in fibrosis
Source: Front Immunol. 2022 Oct 11;13:1004949. doi: 10.3389/fimmu.2022.1004949 (PMC9592691; doi:10.3389/fimmu.2022.1004949)
Supplement: Supplementary file 4 [file Image_2.pdf]

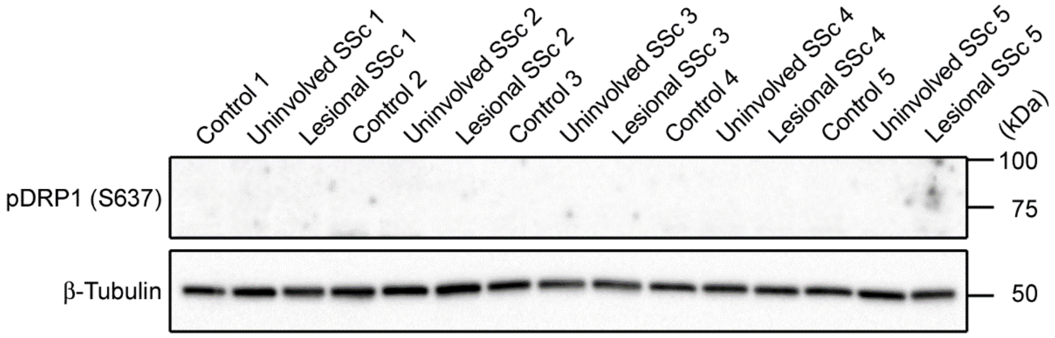

Supplementary Figure S2 | pDRP1 (S637) is undetectable on western blots. Western blot with samples from five control, and five paired uninvolved and lesional SSc fibroblast cultures probed with an antibody against pDRP1 (S637). The blot was reprobed with an antibody against β-tubulin to verify even loading. Migration of protein standards is shown.
