# Supplementary material for: Exploring metabolism in scleroderma reveals opportunities for pharmacological intervention for therapy in fibrosis
Source: Front Immunol. 2022 Oct 11;13:1004949. doi: 10.3389/fimmu.2022.1004949 (PMC9592691; doi:10.3389/fimmu.2022.1004949)
Supplement: Supplementary file 5 [file Image_3.pdf]

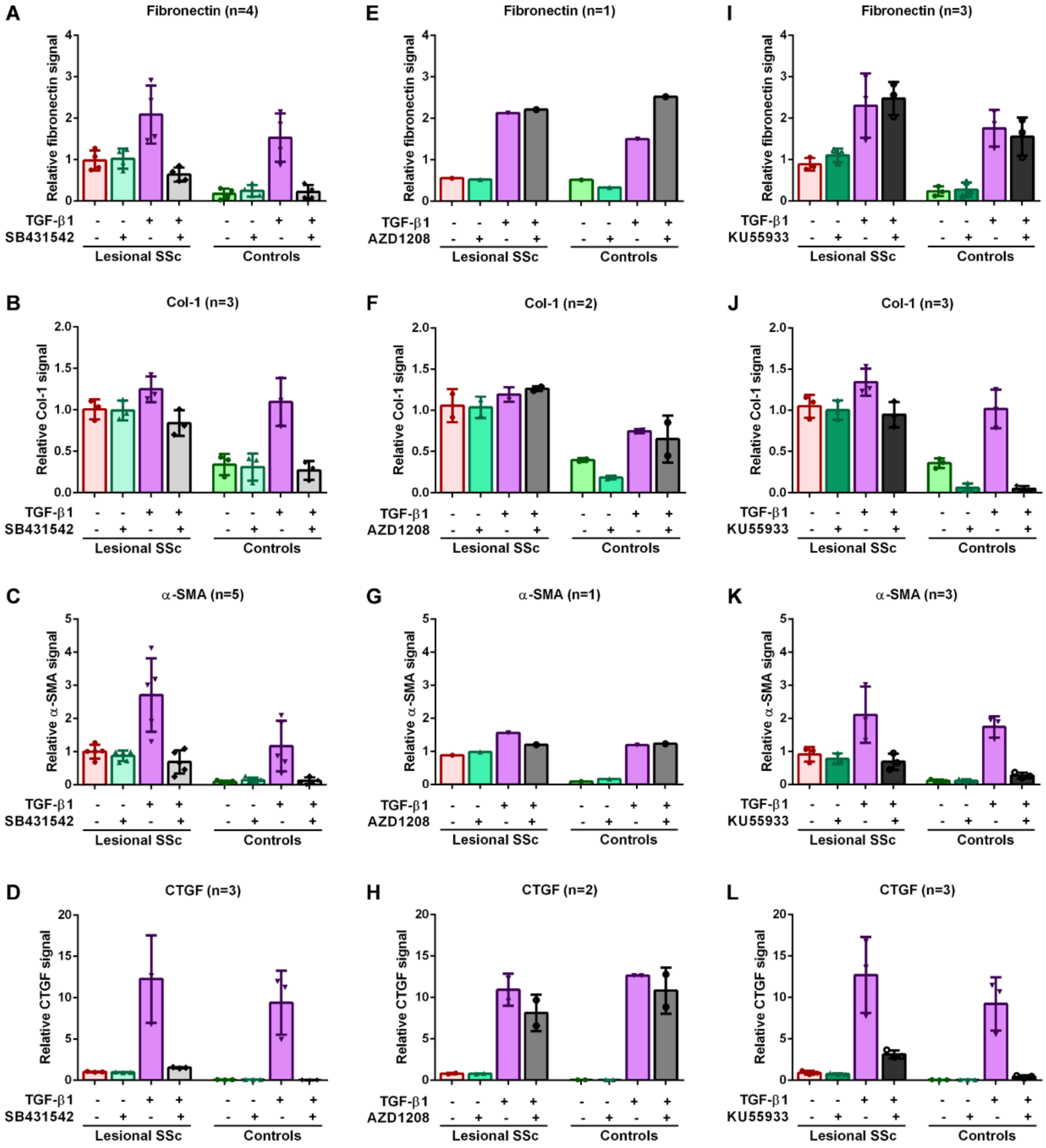


**Supplementary Figure S3** | The kinase inhibitors SB431542 and KU55933, but not AZD1208, prevent TGF-β1-induced overexpression of α-SMA and ECM proteins in lesional SSc and control fibroblasts. Mean western blot signals of the indicated proteins in samples from lesional SSc and control fibroblast cultures treated vehicle or 2 ng/ml of TGF-β1 and/or 10 μM SB431542 **(A–D)**, 20 μM AZD1208 **(E–H)** or 5 μM KU55933 **(I–L)**. Signals were normalized for the β-tubulin signal. Data points show relative values of the individual samples. The number of fibroblast cultures from different donors that were investigated is indicated in each panel. Error bars indicate standard deviations.
